# The Remedial Efficacy of *Spirulina platensis* versus Chromium-Induced Nephrotoxicity in Male *Sprague-Dawley* Rats

**DOI:** 10.1371/journal.pone.0126780

**Published:** 2015-06-01

**Authors:** M. O. Elshazly, Sahar S. Abd El-Rahman, Ashraf M. Morgan, Merhan E. Ali

**Affiliations:** 1 Department of Pathology, Faculty of Veterinary Medicine, Cairo University, Giza, Egypt; 2 Department of Toxicology and Forensic Medicine, Faculty of Veterinary Medicine, Cairo University, Giza, Egypt; National Institutes of Health, UNITED STATES

## Abstract

This study was conducted to investigate the possible protective effect of *Spirulina platensis* against chromium-induced nephrotoxicity. A total of 36 adult male Sprague-Dawley rats were divided into 4 equal groups (Gps). Gp1 served as control, rats of Gps 2, 3, and 4 were exposed to *Spirulina platensis* (300 mg/kg b.wt per os) and sodium dichromate dihydrate (SDD) via drinking water at concentration of 520 mg /l respectively. Chromium administration caused alterations in the renal function markers as evidenced by significant increase of blood urea and creatinine levels accompanied with significant increase in kidney’s chromium residues and MDA level as well as decreased catalase activity and glutathion content in kidney tissue. Histologically, Cr provoked deleterious changes including: vascular congestion, wide spread tubular epithelium necrobiotic changes, atrophy of glomerular tuft and proliferative hyperplasia. The latter was accompanied with positive PCNA expression in kidney tissues as well as DNA ploidy interpretation of major cellular population of degenerated cells, appearance of tetraploid cells, high proliferation index and high DNA index. Morphometrical measurements revealed marked glomerular and tubular lumen alterations. On contrary, spirulina co-treatment with Cr significantly restored the histopathological changes, antioxidants and renal function markers and all the previously mentioned changes as well.

## Introduction

Chromium (Cr) is a heavy metal that is widely distributed in the earth crust. Usually land erosions are the main cause of its release to water, while its major source to air, soil and water is from the fossil fuel combustion and industrial processes [1].

It has several valence states, the most common of which are the hexavalent Cr(VI) and the trivalent Cr(III). The later Cr (III), the chromic form is the predominant form in nature, while the chromate form Cr (VI) is rarely found in nature and is produced particularly from industrial processes [2]. In contrast to Cr III which is an essential trace element, the hexavalent form is more toxic [3–5]. In addition, Potassium dichromate which is a hexavalent form of Cr has been recognized as a human carcinogen and could induce oxidative stress [2, 6]. Chromium has several uses in industry; as a wood preservative, in stainless steel manufacture, leather tanning, paints, as a high temperature catalyst and as a dietary supplement [1].) It has been reported that chromium exposure can results in several toxicity problems such as; dermatotoxicity, neurotoxicity, genotoxicity, cytotoxicity and carcinogenicity [3, 5, 7]. The main known route for chromium excretion is through the kidney [5] with a resultant increase in its chromium content and nephropathy in human and experimental animals [8].

*Spirulina platenesis* is a unicellular cyanbacterium that is full of nutritional wonders and is widely known by its contents of powerful antioxidants and free radical scavenging agents including a potent two phycobiliproteins; phycocyanin and allophycocyanin. That antioxidant activity is prorated mainly to the concentration of phycocyanin [9]. Accordingly, it has been declared that spirulina could amend many toxicity problems induced by heavy metals [10,11], drugs and chemicals [12,13]. It has been proven that Spirulina owns several nutritional and pharmacological properties in addition to its antiviral, anticancer and anti-inflammatory effects, as well as it could regulate lipid and carbohydrate metabolism [14].

### Aim of Work

The main goal of this study was to investigate the possible nephroprotective potential and antioxidant effect of *Spirulina platensis* against chromium-induced oxidative stress and nephrotoxicity in rats.

## Materials and Methods

### Chemicals

Sodium dichromate dihydrate “SDD” (BDH Chemicals Ltd Poole England, Item no 30130). *Spirulina platensis* was obtained as green odorless, water soluble flakes from Arabic Academy for Science, Technology and Maritime Transportation, in Alexandria, Egypt. Its main active principles are phycocyanin and allophycocyanin [9].

### Animals and Experimental Design

36 adult male Sprague-Dawley rats (160-180g) were used in this study. They were purchased from Biological Products & Vaccines Holding Company, Helwan Farm, Cairo, Egypt. Rats were kept in stainless steel cages under standard hygienic conditions (9 rats/cage) and allowed free access to food and distilled water *ad libitum*. All experiments using animals were performed according to the protocol approved by the Intuitional Animal Care and Use Committee at Cairo University. One week post acclimatization, rats were divided into 4 equal groups (Gps) as follow:

Gp 1; served as —ve control, Gp 2; rats exposed to *Spirulina platensis* at 300 mg/kg b.wt dissolved in distilled water daily by oral intubation [11], Gp3; rats exposed to freshly prepared sodium dichromate dihydrate via drinking water at concentration of 520 mg /l (equivalent to 182 mg /l of Cr VI) [15]. The prepared solution was the sole solution of drinking for rats. Gp 4; rats exposed to SDD concomitantly with *Spirulina platensis* with the same previously mentioned doses and routes.

Careful observation for all animals of all groups was carried out along the experimental period (3 months) and any clinical abnormalities were recorded. At the end of the experimental period, all animals were weighted and anesthetized using a mixture of equal volumes of (diethyl ether, chloroform and acetone) then animals were sacrificed for samples collection. Two blood samples were collected from each animal of all groups, one without anticoagulant for measurement of serum urea and creatinine levels and the other on heparin for determination of Cr residue in blood.

In addition, kidneys were immediately dissected out of the body, wiped off blood and weighted. Part of the kidney was stored at -20°c for determination of Cr residues and oxidative stress markers in its tissue homogenate. The other part was kept in buffered neutral formalin 10% for histopathological studies.

### Serum Biochemical Analysis

Renal function markers; urea [16] and creatinine [17] were assessed in serum samples of rats of all groups.

### Evaluation of Antioxidant Markers in Kidney Tissue Homogenate

Malondialdehyde level [18], catalase activity [19] and GSH contents [20] were assessed in kidney tissue homogenates of all animals.

### Estimation of Chromium Residues in Blood and Renal Tissue

Chromium residues were determined in both blood and kidney tissue homogenates of all rats’ groups by Atomic Absorption Spectrophotometer (AAS, unicam 969) according to [21].

### Histopathological and Histochemical Studies

Formalin fixed kidney tissues were routinely processed using conventional paraffin embedding technique. Sections of about 4–5 μm were stained with H&E [22].

PAS stain, Feulgen’s reaction (for DNA ploidy interpretation) and Azan stain (for fibroblastic proliferation) were used for histochemical investigations on need [22].

### Histomorphometry

For morphometrical study, H&E stained kidney sections of each animal were used. Two sections per slide and 10 non-overlapping microscopic fields were studied. Kidney measurements included glomerulus area, glomerulus diameter, Bowman's space and the tubular lumen area were all interpreted. A computerized microscopic image analyzer attached for full HD microscopic camera (Leica microsystems, Germany) was used for image analysis and morphometrical data acquisition.

### DNA Ploidy Interpretation

For demonstrating DNA content, sections were stained with Feulgen’s reaction to demonstrate DNA as a magenta color. The distribution of a population of cells within the cell cycle was cytometrically analyzed using Leica Qwin 500 Image Analyzer (LEICA Imaging Systems Ltd, Cambridge, England,).

### Immunohistochemical Studies

Detection of proliferating cell nuclear antigen (PCNA) expression on kidney’s paraffin sections of selected control and treated rats using avidin-biotin Peroxidase (DAB, Sigma Chemical Co.) was done according to method described by [23]. Tissue sections were incubated with a monoclonal antibody to PCNA (Dako Corp, Carpenteria, CA) and reagents required for the avidin-biotin peroxidase (Vactastain ABC peroxidase kit, Vector Laboratories) method for the detection of the antigen—antibody complex. PCNA expression was localized by the chromagen 3,3-diaminobenzidine tetrahydrochloride (DAB, Sigma Chemical Co.).

### Statistical Analysis

The obtained data are presented as mean ± SE. Statistical significance between the different groups was analyzed using ANOVA test (SPSS: statistical package for social sciences 10.0 for windows) followed by Duncan’s Multiple Range Test [24].

## Results

Results of the present study revealed that Cr administration to rats induced deleterious effects in the renal histology, which was reflected on the examined renal function markers and the natural antioxidant status.

### Body Weight and Kidney Weight Index

**Fig 1:**
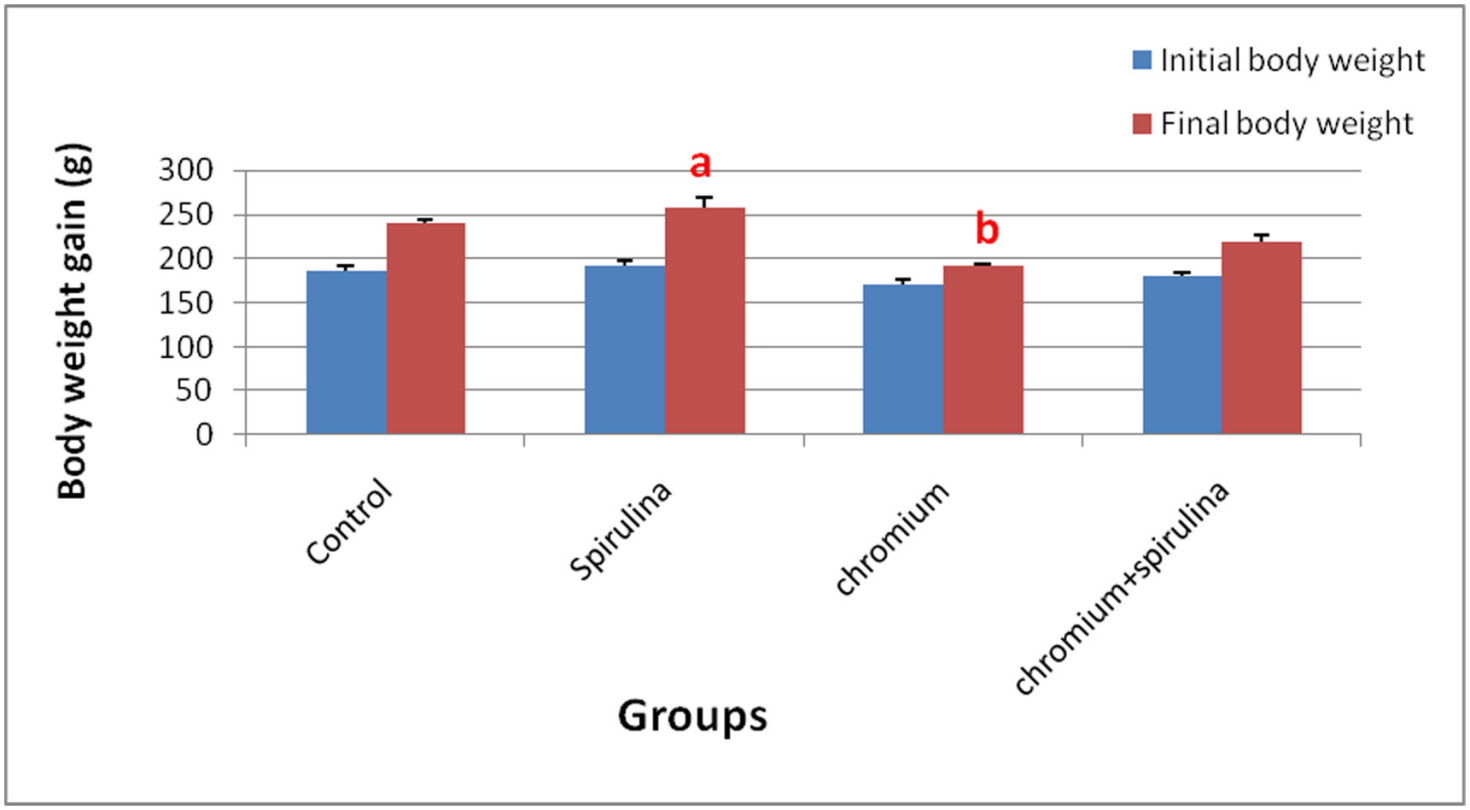
The effect of SDD, spirulina and their co-administration on body weight gain of all treated groups.

The body weight of chromium-treated rats was significantly decreased as compared to that of control group (Fig 1). On the other hand, the co-treatment with spirulina significantly conserved the body weight gain. Surprisingly, rats treated with sole spirulina showed significant increase in their body weight gain in comparison to that of controls.

**Fig 2:**
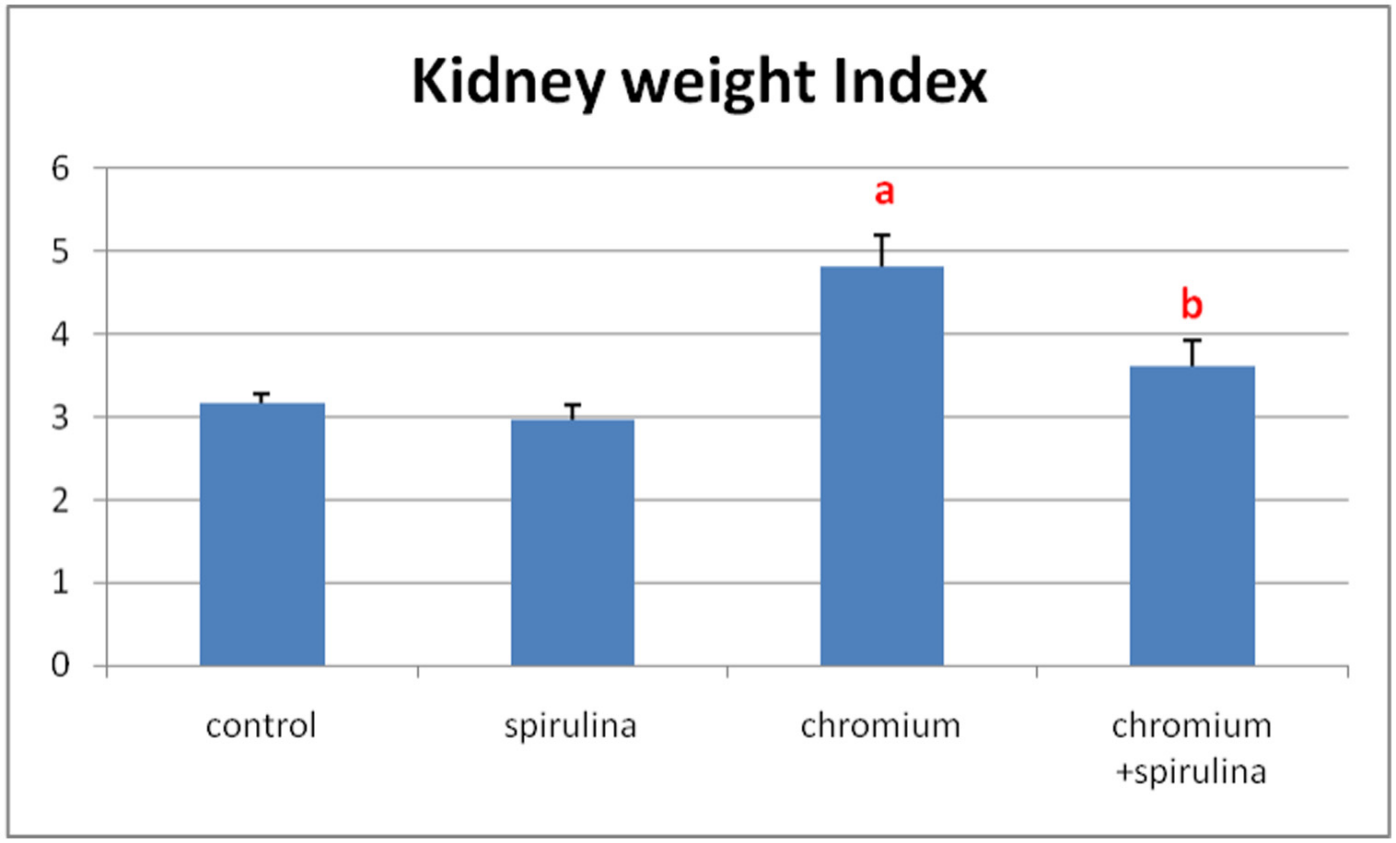
The effect of SDD, spirulina and their co administration on kidney weight index of all treated groups.

The kidney weight index (final kidney weight / final body weight *100) of Cr treated group showed significant increase compared to that of the control group. However, the co-treatment with spirulina ameliorated that increase in kidney index and conserved it near to control values (Fig 2).

### Biochemical Parameters and Oxidative Stress Markers

**Fig 3:**
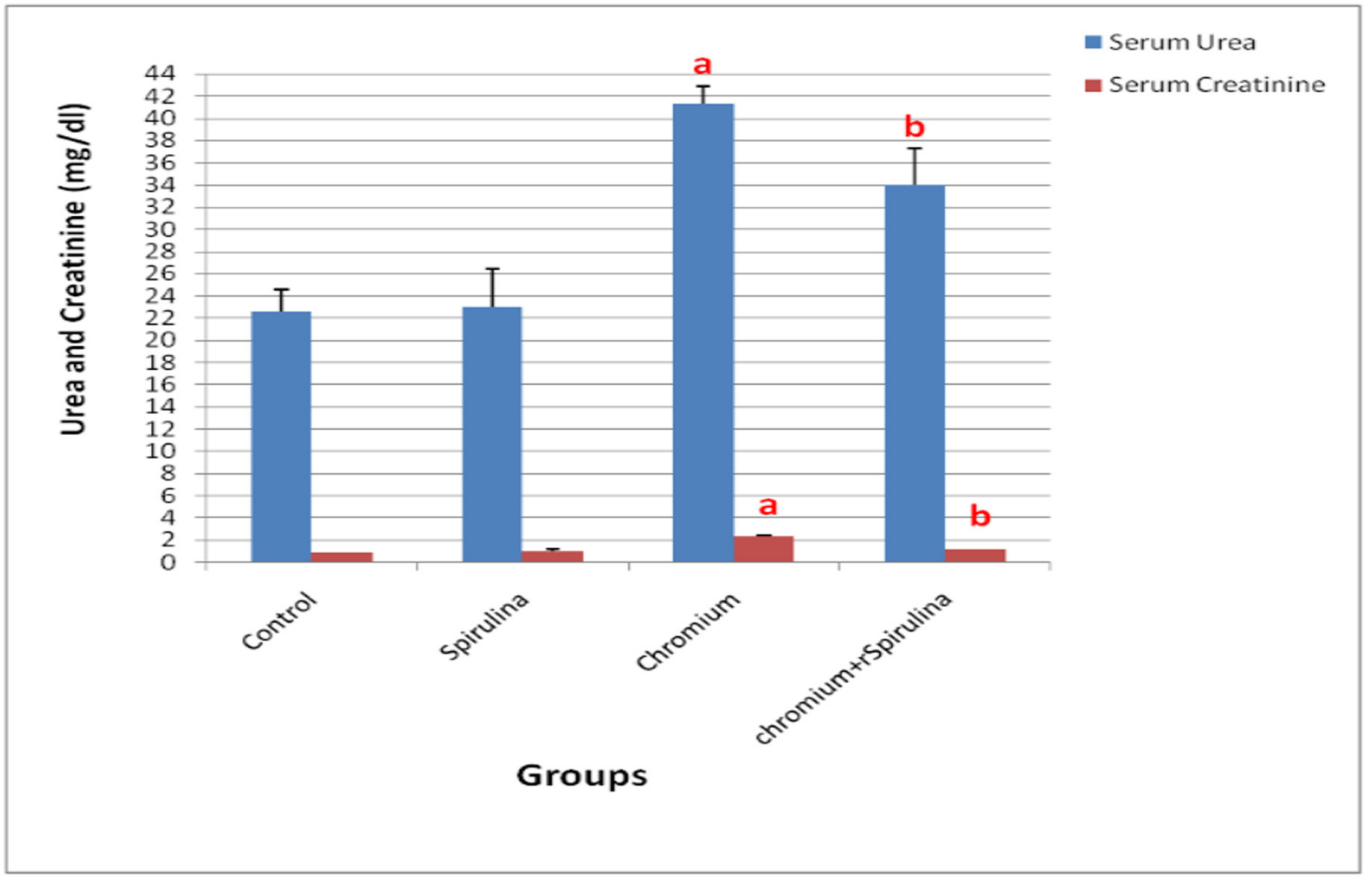
The effect of SDD, spirulina and their co administration on serum urea and creatinine levels of all treated groups.

A significant elevation in renal function markers; serum urea and creatinine levels was observed in Cr treated rats compared with those of control groups (Fig 3). It was shown that the co- administration with spirulina brought about a significant restorative reduction of the elevated serum urea and creatinine levels versus those measured in chromium treated group.

**Fig 4:**
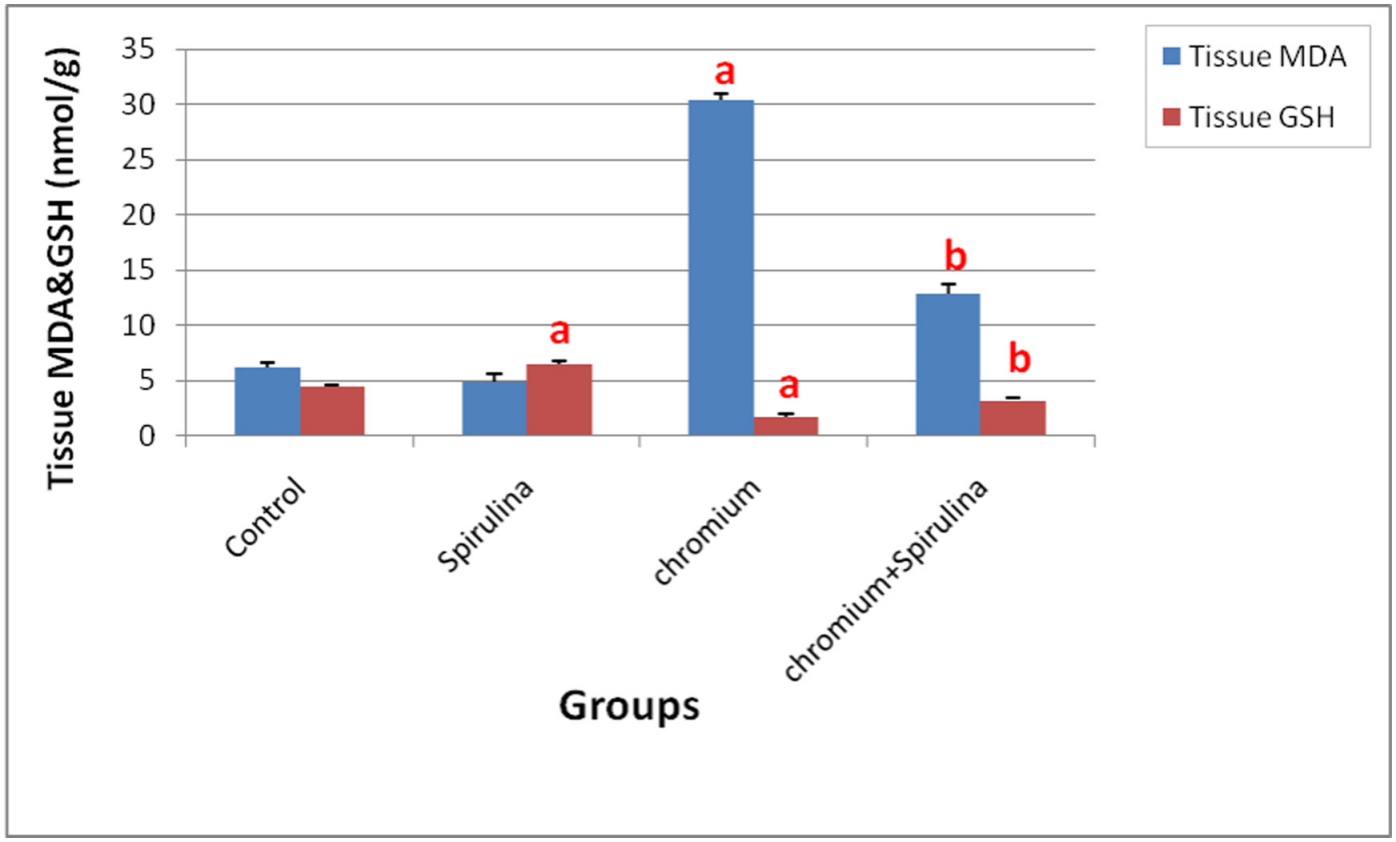
The effect of SDD, spirulina and their co administration on MDA and GSH contents in kidney tissue of all treated groups.

Concerning the oxidative stress markers levels, a significant elevation of renal MDA content was detected in chromium-exposed rats (Fig 4).

**Fig 5:**
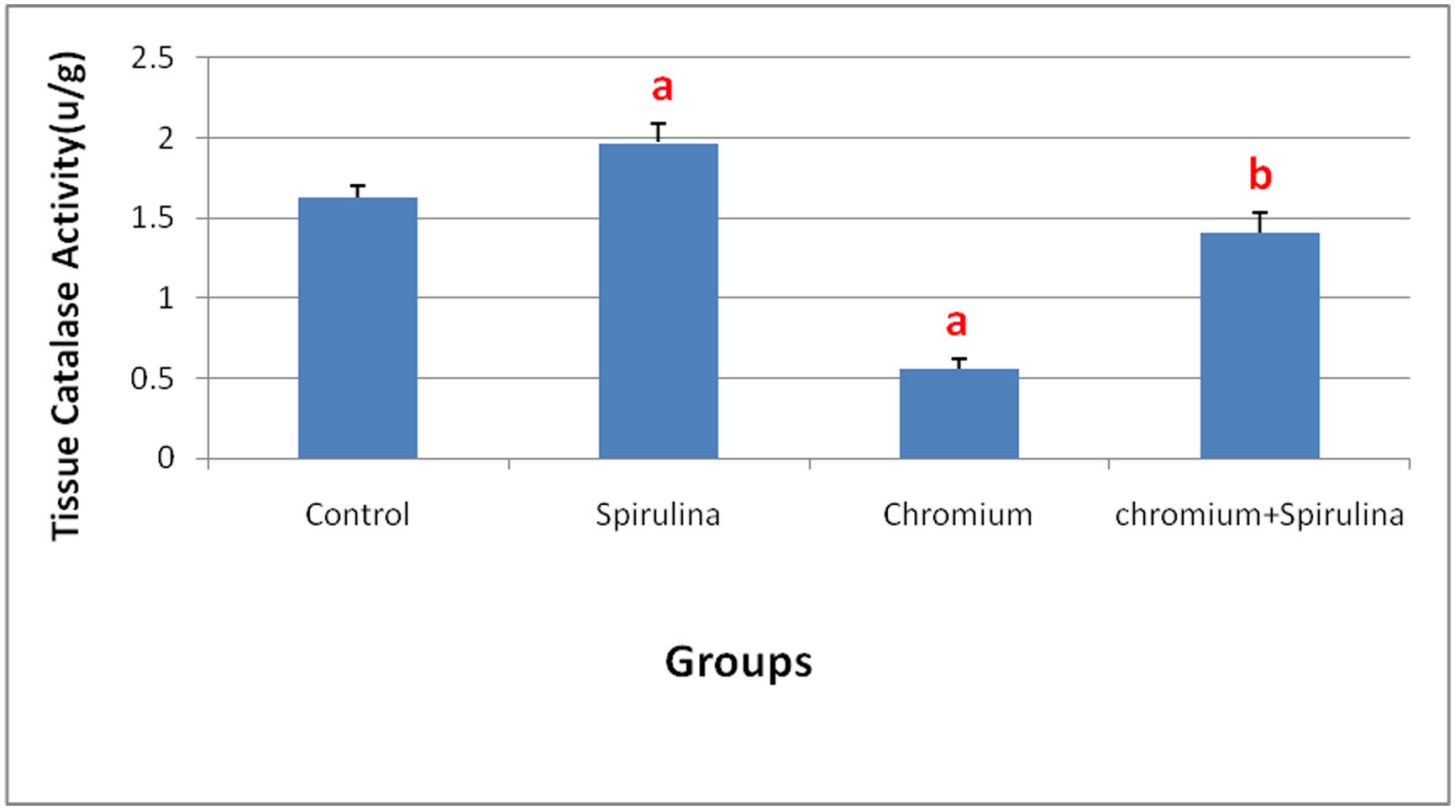
The effect of SDD, spirulina and their co administration on catalase activity in kidney tissue of all treated groups.

On the other hand, renal GSH content and catalase activity were significantly reduced following Cr administration (Fig 5). However, the spirulina co-treatment significantly retrieved those altered levels of the antioxidants. Moreover, the sole administration of spirulina did not influence their values.

### Chromium Residues in Blood and Renal Tissue

**Fig 6:**
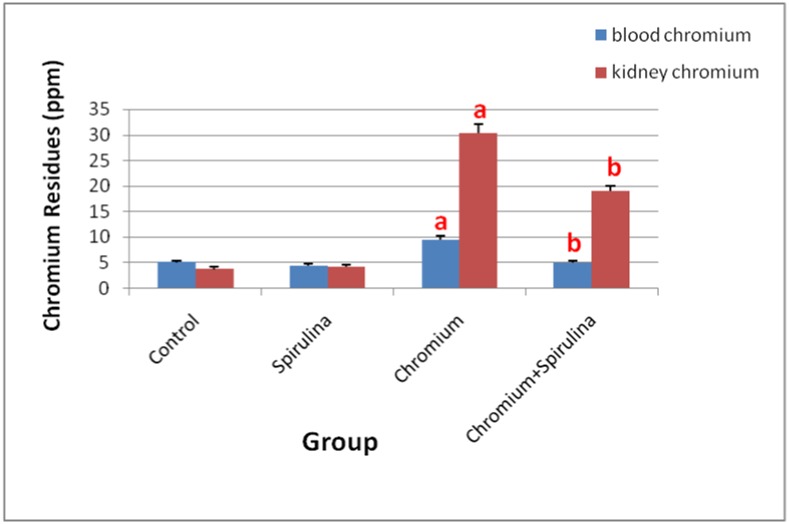
The effect of SDD, spirulina and their co-administration on chromium contents in blood and kidney tissue.

Atomic absorption spectrophotometer assay of Cr residue in blood and kidney tissue homogenates of all groups’ revealed significant increase in Cr content in both blood and renal tissue of Cr treated rats compared with that of controls. Both of the control-ve and spirulina treated groups showed traces of Cr in their blood and their renal tissue homogenates (Fig 6). Over and above, the co-treatment with spirulina significantly decreased those elevated levels of Cr residues in blood and the renal tissue samples as well.

### Results of Histopathological Examination

**Fig 7:**
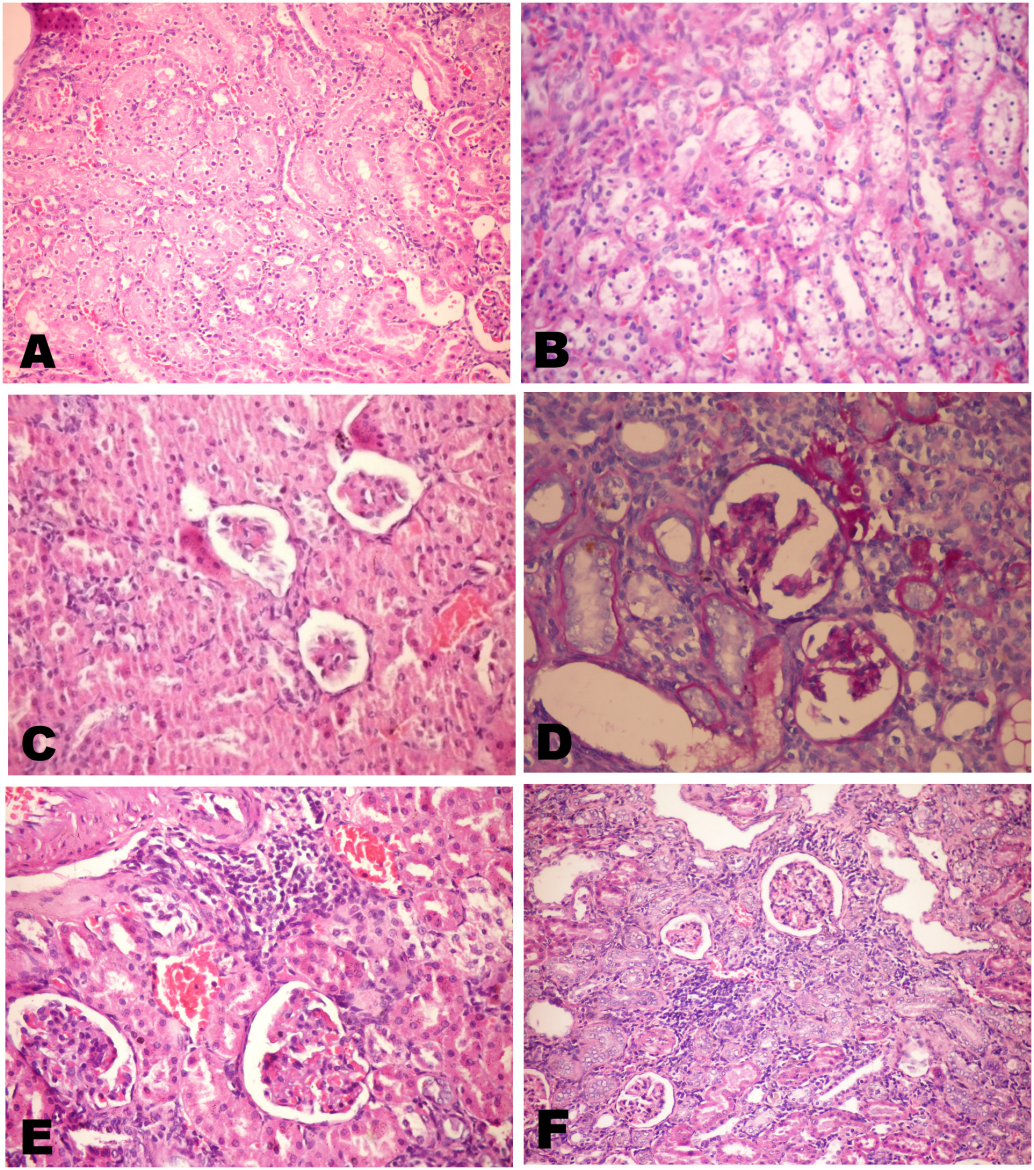
Kidneys of chromium treated rats. Showing A) Swelling of the tubular lining epithelium with variable degrees of granular and vacuolar degeneration and single cell necrosis(H&E X400). B) Wide spread nuclear pyknosis among the tubular epithelium (H&E X400). C) Atrophy of the glomerular tufts with mesangeal necrosis (H&E X400). D) Thickening of the glomerular basement membrane (PAS X400). E) Interstitial nephritis, notice the congested blood vessels and focal mononuclear inflammatory infiltrates (H&E X400). F) Proliferative hyperplasia of the renal tubular epithelium that appeared basophilic with vesicular and basophilic nuclei (H&E X200).

Microscopical examination of different kidney sections from control and spirulina treated groups' revealed normal histological structure. While, examination of various sections of Cr-treated rats' revealed marked renal alterations. An evident congestion of the interstitial and glomerular blood vessels with occasional appearance of inter tubular pockets of hemorrhage were observed. All Cr-exposed rats showed more or less the same tubular changes (Fig 7A) including cytoplasmic swelling, variable degrees of granular and vacuolar degeneration and marked necrosis of the tubular epithelium either as single cell necrosis or as massive necrosis. The necrotic cells appeared either as homogenous structureless esinophilic masses without any nuclear structure or appeared with wide spread nuclear pyknosis (Fig 7B). Granular or homogenous renal cast was observed in the lumina of some tubules. These tubular changes were more conspicuous in the cortex and the outer stripe of the outer medulla. The renal glomeruli revealed thickening of the glomerular basement membrane, mesangeal necrosis, shrinkage or atrophy of the glomerular tufts with thickening of the parietal layer of the Bowman's capsule (Fig 7C and 7D). The later appeared either as scattered glomeruli or as foci. In most cases, marked focal interstitial nephritis (Fig 7E) was evident which were occasionally accompanied with mild to moderate interstitial scarring.

The unique lesion noticed with variable degrees of severity was; an obvious proliferative hyperplasia of the renal tubular epithelium that appeared basophilic with vesicular and basophilic nuclei with great atypia (Fig 7F).

**Fig 8:**
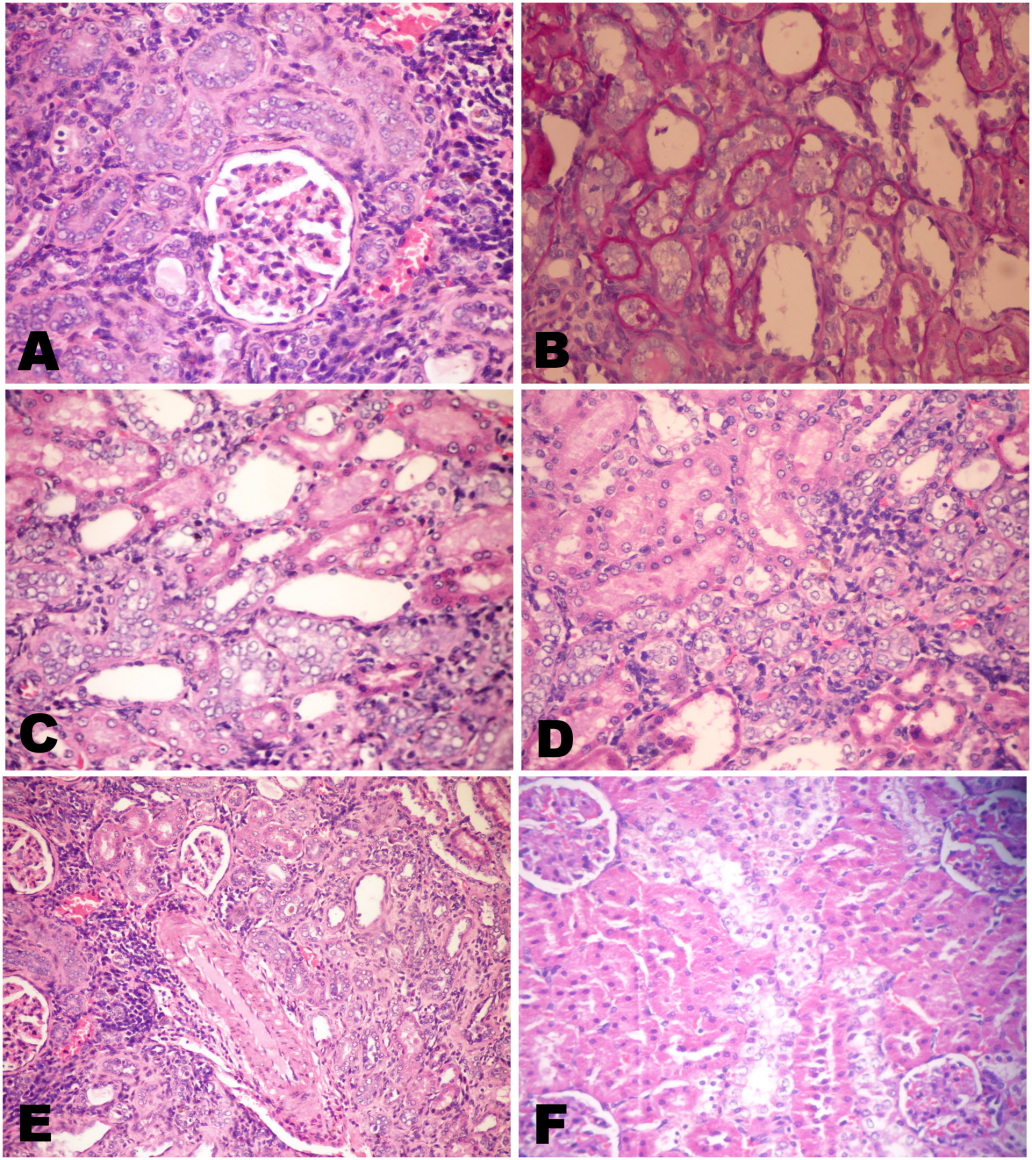
A-E Kidneys of chromium treated rats. Showing A, B) Scattered foci of hyperplastic tubules with thick basement membrane (H&E and PAS X400). C, D) The hyperplastic cells partially lined the tubular circumference or away around the tubule insinuating in the intertubular areas as rows (thick arrow) or clumps (arrow) or forming a tubular pattern (H&E X400). E) Mononuclear inflammatory cells infiltration accompanying the hyperplastic tubules with minimal increased number of mesenchymal interstitial cells (H&E X400). F) Kidney of chromium and spirulina co-treated rats showing wide spread foci of regenerative tubules (H&E X200).

The hyperplastic tubules appeared either as scattered foci of tubules with thick basement membrane (Fig 8A and 8B) or extensively spread along the parenchyma. Characteristically, the hyperplastic cells often only either partially lined the tubular circumference or in other parts away around the tubule insinuating in the intertubular areas as clumps or rows or forming a tubular pattern (Fig 8C and 8D). Overwhelmingly, that hyperplasia was associated with mononuclear inflammatory cells infiltration and a minimal increased number of mesenchymal interstitial cells (Fig 8E) but, only rarely with fibroplasia occurrence.

It is worth mentioning that; the co-treatment with spirulina conspicuously exhibited a protective role and markedly reduced tissue damage induced by chromium. Neither hyperplasia nor fibroplasia was observed in the spirulina co-treated group. Often only a mild to moderate degrees of degenerative changes among the tubular linings, marked decrease in the cellular necrosis along with wide spread foci of regenerative tubules (Fig 8F) and absence of the glomerular changes.

### Histomorphometrical Aspects

Chromium-exposed rats showed increased number of the affected glomeruli. Data obtained from kidney histomorphometrical measurements elucidated statistically significant decrease in the mean values of glomerulus area, glomerulus diameter, the proximal and distal convoluted tubules lumen area as well as significant increase of Bowman’s space of chromium treated rats in comparison with their control group (Tables 1 and 2). On contrary, spirulina co-treatment resulted in statistically significant restorative effect on the altered glomerular and tubular measurements.

**Table 1:** Values of the histomorphometrical measurements of glomerulus area, glomerulus diameter and Bowman's space of control and treated groups.

Group	Parameter		
	Glomerulus area (sq μm)	Glomerulus diameter (μm)	Bowman's Space (sq μm)
**Control**	2215.22 ± 94.1	49.8 ± 3.1	424.21 ± 35.22
**Spirulina treated group**	2195.73 ± 106.3	50.06 ± 3.07	423.71± 23.38
**Chromium treated group**	967.7 ± 98.2 ^a^	34.4±1.98 ^a^	794.96 ± 89.22 ^a^
**Chromium and spirulina co-treated group**	1866 ± 138.4 ^b^	47 ± 2.1 ^b^	381.85 ± 50.70 ^b^

Values are expressed as Means± SE.

^a^: Significantly different from corresponding control group at p≤ 0.05.

^b^: Significantly different from corresponding Cr alone treated group at p≤ 0.05.

**Table 2:** Values of the histomorphometrical measurements of tubules lumen area of control and treated groups.

Group	Parameter	
	PCT Lumen (sq μm)	DCT Lumen (sq μm)
**Control**	83.48 ± 7.11	119.39 ± 15.56
**Spirulina treated group**	80.1 ± 6.4	118.82 ± 14.7
**Chromium treated group**	43.2 ± 2.5 ^a^	93.2 ± 5.9
**Chromium and spirulina co-treated group**	55.7 ± 2.1 ^b^	96.87 ±0.91

Values are expressed as Means± SE.

^a^: Significantly different from corresponding control group at p≤ 0.05.

^b^: Significantly different from corresponding Cr alone treated group at p≤ 0.05.

### DNA Ploidy Interpretation

The cytometrical analysis of DNA contents of negative and spirulina treated groups revealed that; around 70% of the cells examined for DNA ploidy were diploid (content of DNA = 2C) and within normal value of hypoploid cells (DNA content less than 1.5 C). The proliferation index of control groups was of medium value and the DNA index was 1.000.

On the other hand, chromium treated group showed that the major cell population was degenerated cells (29.5%) with low normal diploid cells (38.077) as well as the appearance of (2.923%) tetraploid cells (DNA content = 4C) which was not present in control groups. The proliferation index of chromium treated rats was of high value and the DNA index was increased (1.375). Spirulina co-treatment resulted in decreasing the population of the degenerated cells (23.70%), restoration of diploid cells to near the normal value (57.762%) with medium proliferation index and decreased DNA content (1.15) compared with those of the sole chromium exposed group (Table 3).

**Table 3:** The cytometrical analysis of DNA content and distribution of the population of cells within the cell cycle of control and treated groups.

Group	Parameter				
	Hypoploid cells (%)	Diploid cells (%)	Proliferation index (%)	Tetraploid cells (%)	DNA index (%)
**Control**	12.745	70.5888	16.667	0.0	1.000
**Spirulina**	11.8	71.5	16.7	0.0	1.000
**Chromium**	37.5	38.007	21.5	2.923	1.370
**Chromium and spirulina co-treated group**	23.70	57.762	16.667	0.0	1.15

### Immunohistochemistry

**Fig 9:**
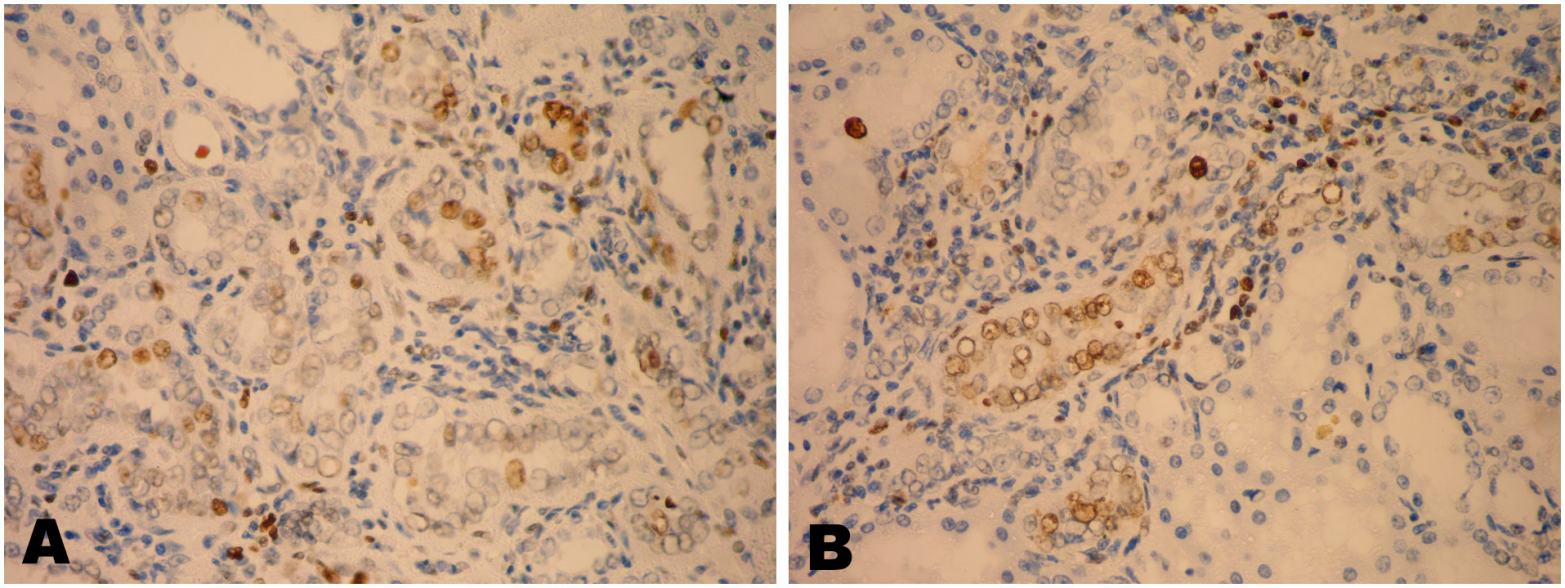
A, B) Kidney of chromium treated rat showing positivity for PCNA expression in the nuclei of the hyperplastic tubular epithelial cells (X400).

Widely scattered tubular cell nuclei were positive for PCNA expression in kidney sections of Cr-exposed animals particularly the foci of altered tubular cells that showed proliferative hyperplasia (Fig 9A and 9B). However, there was no recognizable increase in PCNA positivity throughout the kidney sections of control and spirulina co-treated groups.

## Discussion

The present study showed that chromium exposure provoked deleterious effects on the renal tissue with loss of the functional integrity of the kidney as was evidenced by the observed histological and biochemical alterations in addition to altered levels of oxidative stress markers. These results are in accordance with those reported by [25–27]. Nephrotoxicity in human and experimental animals could be induced by Cr (VI) compounds [28]. In addition it was stated that the target organ for systemically absorbed chromate is the kidney and the resultant nephrotoxicity or the complete renal shut down could be the primary cause of death to acute chrome exposure [28].

The present work revealed a significant increase in chromium residues in blood and renal tissue in Cr-treated group compared with the other groups. It is renowned that the kidney is the fundamental route of Cr excretion with an increase in kidney chromium content upon acute exposure to potassium dichromate in rats [5]. In humans, the absorbed chromium is excreted primarily via urine. It was found that the half-life for elimination of chromium when given as potassium chromate (0.05 mg chromium (VI) kg-1 in drinking water) is estimated to be approximately 35–40 hours [4]. In the present work, Cr induced significant decrease in body weight gain, which could be attributed to the direct action of Cr which is in agreement with results of **Dey and Roy** [21]. The former was accompanied with significant increase in kidney weight index as a result of increased kidney weights which could be related to the observed pathological alterations in the renal parenchyma. The observed tubular changes, glomerulonephritis and focal interstitial nephritis in the present work are harmonious with those mentioned by [8, 28–30]. The later lesions were associated with histomorphometrical glomerular and tubular changes, which are in agreement with those mentioned by **Abdel-Rahman et al.,** [31] who pointed out the adverse effects of chromium on the histomorphometrical parameters of rabbit’s kidney. Such morphometrical changes could be attributed to the observed wide spread atrophy of the glomerular tufts that resulted in increased Bowman's capsule area. Whereas the tubular changes may be related to cellular swelling, degeneration and tubular obstruction by cell debris. Furthermore, **Soudani et al.,** [27] attributed tubular degeneration and damage due to Cr toxicity to accumulation of inflammatory cells associated with chromium toxicity. The altered histological picture in the present work was confirmed by the altered biochemical results of increased level of renal functional markers (urea and creatinine) which may be due to dysfunction of cell membrane permeability, toxic injuries of tubules and tubular obstruction by cell debris, which reflected on loss of functional integrity in the kidney that comes in accordance with [25]. Regarding the observed proliferative hyperplasia of the renal tubular epithelial cells in the present study, it may be due to the direct irritative effect of Cr on the cells after absorption and during excretion. The former hyperplasia was accompanied with marked cellular atypia, inflammatory infiltrates and mild to moderate fibroplasia as well as marked PCNA positivity among those hyperplastic cells throughout the kidney sections. The DNA ploidy interpretation of kidney sections of the later cases that showed hyperplasia revealed high proliferation (21.5) and DNA (1.375) indices, a result which is in agreement with **Wang et al.,** [32] who mentioned that the DNA index of 1.0 indicates normal diploid cells in the G0/G1 phase while, some cell populations such as cancer cells can have abnormal DNA content. Accordingly, the detected DNA index in kidney sections with hyperplasia indicates abnormal higher DNA content. The proliferation index is automatically expressed as the percentage of cells engaged in the S- phase of the cell cycle and it was classified into low (< 10%), medium (10–20%) or high (> 20%) [33]. Hence the observed proliferation index in our results is an indication of increased number of S-phase engaged cells in kidney sections that showed hyperplasia.

That hyperplasia could elucidate a precarcinogenic state particularly as it was accompanied with high degree of cellular atypia and obscure arrangement of renal tubules in the altered foci. **Ozaki et al.,** [34] supported PCNA evidence of hyperplasia in renal tissue induced by one of the peroxisome proliferators.

In addition, the present work revealed that Cr administration altered the levels of oxidative stress markers as detected by increased level of MDA; a lipid peroxidation marker that is a good evidence for this oxidative stress as well as decreased activity of catalase and renal glutathione content as compared with the normal control and the co-treatment groups as well. The role of oxidative stress in dichromate-induced kidney damage has been supported by the present work and previous studies [8, 29, 35–37]. Those results were strengthened by others which declared that the exposure of female rats to K_2_Cr_2_O_7_ for 21 days induced renal damage and disturbed the oxidative stress markers with a significant increase in kidney malondialdehyde, superoxide dismutase, plasma creatinine, and uric acid levels, accompanied with significant decrease in catalase, glutathione peroxidase, non-protein thiol, Metallothionein and plasma urea levels [26].

It is well known that, several body systems can perform reduction process of the unstable chromium (VI) to chromium (V), chromium (IV), and ultimately to chromium (III) by endogenous substances such as ascorbate and glutathione and it is believed that this process generates free radicals aiding chromium toxicity and resulting in damage of the cellular components such as cellular proteins, lipids, and DNA leading to oxidative stress and inducing soft tissues damage such as liver, pancreas, cerebellum and kidney [4]. Meanwhile, it was concluded by [3, 28] that, renal damage induced by chromium may come from the generation of toxic radicals during reduction of hexavalent chromium to trivalent one inside the cell. Following potassium dichromate exposure, most of the anti-oxidant enzymes become inactive either as a result to the direct binding of heavy metals to SH group of the enzyme active site or to the displacement of metal co-factors from active site [8].

The role of antioxidants in protecting the cells is well known. In the present work, the co-administration of spirulina to chromium conspicuously restored the renal biomarkers parameters (reduced the elevated serum urea and creatinine levels) and the antioxidant enzyme activities that was reflected on the good histological picture. The restoration effect of spirulina could be due to its potential antioxidant properties which improved the renal function following its co-treatment with SDD mediated via the attenuation of SDD-induced oxidative stress and the modulation of the damaged renal tissue particularly the glomerular filtration [38]. Hence, our results clarified the ability of the used antioxidant in protection against the Cr-induced renal toxicity. The protective effects of spirulina have been reported against renal injury induced by gentamicin [39,40] as well as oxalate [41,42], sodium fluoride-induced oxidative alterations in offspring of pregnant rats [43] and lead-induced brain damage in newborns [44]. The aforementioned nephropotective effect of spirulina could be attributed to its great antioxidant contents which act by inhibiting lipid peroxidation, scavenging free radicals and promoting the activity of the enzymatic free radicals scavengers in the cells. Moreover, owing to its content of antioxidants active constituents such as c-phycocyanin, carotenoids, vitamins, minerals, lipids, proteins and carbohydrates, Spirulina could be used to prevent and treat hepatic and renal diseases especially those induced by oxidative damage [45,46]. The metalloprottective role of spirulina may be attributed to its antioxidant components such as β-carotene, selenium and others that may scavenge free radicals generated by chromium with a consequent reduction of cell damage, especially damage to DNA molecule thus playing a role in the repair of the regeneration process of the damaged cell [47].

## Conclusion

Our results revealed that oxidative stress plays a major role in chromium-induced nephrotoxicity. Many previous interventions have proven the effectiveness of antioxidants in ameliorating chromium-induced toxicity. Over and above, our results declared that *Spirulina platensis* is a potent antioxidant that could ameliorate the effect of chromium-induced nephrotoxicity and provided a near complete protection in terms of tissue and biochemical changes, antioxidant markers activity and oxidative stress as well.
